# Metabolomic analysis of *Mycobacterium tuberculosis* reveals metabolic profiles for identification of drug-resistant tuberculosis

**DOI:** 10.1038/s41598-023-35882-2

**Published:** 2023-05-27

**Authors:** Pratchakan Chaiyachat, Benjawan Kaewseekhao, Angkana Chaiprasert, Phalin Kamolwat, Ditthawat Nonghanphithak, Jutarop Phetcharaburanin, Auttawit Sirichoat, Rick Twee-Hee Ong, Kiatichai Faksri

**Affiliations:** 1 Department of Microbiology, Faculty of Medicine, Khon Kaen University, Khon Kaen, Thailand; 2 Research and Diagnostic Center for Emerging Infectious Diseases (RCEID), Khon Kaen University, Khon Kaen, Thailand; 3 Office for Research and Development, Faculty of Medicine Siriraj Hospital, Mahidol University, Bangkok, Thailand; 4 Bureau of Tuberculosis, Department of Disease Control, Ministry of Public Health, Nonthaburi, Thailand; 5 Department of Systems Biosciences and Computational Medicine, Faculty of Medicine, Khon Kaen University, Khon Kaen, Thailand; 6 Saw Swee Hock School of Public Health, National University of Singapore and National University Health System, Singapore, Singapore

**Keywords:** Computational biology and bioinformatics, Microbiology

## Abstract

The detection of pre-extensively (pre-XDR) and extensively drug-resistant tuberculosis (XDR-TB) is challenging. Drug-susceptibility tests for some anti-TB drugs, especially ethambutol (ETH) and ethionamide (ETO), are problematic due to overlapping thresholds to differentiate between susceptible and resistant phenotypes. We aimed to identify possible metabolomic markers to detect *Mycobacterium tuberculosis* (*Mtb*) strains causing pre-XDR and XDR-TB. The metabolic patterns of ETH- and ETO-resistant *Mtb* isolates were also investigated. Metabolomics of 150 *Mtb* isolates (54 pre-XDR, 63 XDR-TB and 33 pan-susceptible; pan-S) were investigated. Metabolomics of ETH and ETO phenotypically resistant subgroups were analyzed using UHPLC-ESI-QTOF-MS/MS. Orthogonal partial least-squares discriminant analysis revealed distinct separation in all pairwise comparisons among groups. Two metabolites (meso-hydroxyheme and itaconic anhydride) were able to differentiate the pre-XDR and XDR-TB groups from the pan-S group with 100% sensitivity and 100% specificity. In comparisons of the ETH and ETO phenotypically resistant subsets, sets of increased (ETH = 15, ETO = 7) and decreased (ETH = 1, ETO = 6) metabolites specific for the resistance phenotype of each drug were found. We demonstrated the potential for metabolomics of *Mtb* to differentiate among types of DR-TB as well as between isolates that were phenotypically resistant to ETO and ETH. Thus, metabolomics might be further applied for DR-TB diagnosis and patient management.

## Introduction

Tuberculosis (TB) is a major infectious disease caused by *Mycobacterium tuberculosis* (*Mtb*)^1^. Treatment of TB is becoming more difficult and challenging with the emergence of drug resistance. In 2020, 25,681 cases were reported of pre-extensively drug-resistant tuberculosis (pre-XDR-TB; resistant to isoniazid/rifampicin and any fluoroquinolone) or XDR-TB (resistant to rifampicin, plus any fluoroquinolone, plus bedaquiline (BDQ) and/or linezolid (LZD))^1^. The global treatment success rate for DR-TB remains low, at 59 percent^2^. In particular, the treatment success rate for XDR-TB was only 39%^1^. Selection of appropriate anti-TB drugs for treatment of each patient requires evaluation of the drug-resistance properties of their strain of *Mtb*: this is crucial for DR-TB treatment^3^.

Improper diagnosis of DR-TB not only raises the chance of drug resistance developing, but it can also result in lower therapeutic efficacy, increased side effects, and decreased patient compliance. Furthermore, according to a prior epidemiological investigation, the recurrence rate of DR-TB is around 61.3%, which is significantly higher than for drug-susceptible TB (27.9%)^4^. Transmission of primary DR-TB, especially pre-XDR and XDR-TB^5,6^ is a serious situation requiring novel tools for detection and classification. Therefore, the investigation of specific markers to rapidly identify DR-TB, especially pre-XDR/XDR-TB, is essential for early diagnosis and timely drug-regimen adjustment in patients.

Metabolomics, an emerging science of the “-omics” era, can be used in the identification and quantification of low-molecular-weight metabolites (< 1500 Da). Metabolic fingerprints can potentially be used to discriminate between states of health and disease^7^. In TB, metabolomics analysis of *Mtb* isolates is one such form of precision medicine offering personalized management of TB patients. Recently, metabolomics has been applied to generate metabolite patterns that can differentiate among DR-TB types^8^. Key metabolites associated with specific forms of TB disease have the potential for use as diagnostic biomarkers or indicators^9,10^. Therefore, this approach could strengthen the performance of drug-susceptibility testing (DST) and can also be used in personalized medicine for TB patient management. However, the number of studies applying this technology is limited.

For DST, discrepancies between genotypic and phenotypic test results can occur^11–14^. Determination of resistance status against some anti-TB drugs, especially ethambutol (ETH) and ethionamide (ETO), can be problematic due to overlapping minimum inhibitory concentration (MIC) thresholds to differentiate between susceptible and resistant genotypes^5^. Only 38% of isolates phenotypically resistant to ETO (and 35% for ETH) were also genotypically resistant^5^. In particular, there was an 80% disagreement for ETO between genotypic DST and the phenotypic test provided by the Mycobacteria Growth Indicator Tube (MGIT)^15^. Metabolomic analysis might provide additional markers to help resolve such discrepancies.

Here, we aimed to determine the metabolomic profiles of *Mtb* using ultra-high performance liquid chromatography coupled with the electrospray ionization-quadrupole-time of flight-mass spectrometry (UHPLC-ESI-QTOF-MS/MS) approach to distinguish pre-XDR and XDR-TB isolates from drug-susceptible isolates. We also determined the specific metabolites present in ETO- and ETH-resistant isolates of *Mtb*.

## Results

### Sample characteristics

One hundred and fifty *Mtb* isolates were included for metabolomic analysis. These comprised 54 pre-XDR and 63 XDR-TB isolates identified as such based on the previous WHO definitions^1^. Thirty-three pan-S isolates were used as the control group. Phenotypic DST results for all isolates were available for 14 anti-tuberculosis drugs (isoniazid (INH), rifampicin (RIF), streptomycin (STM), ethambutol (EMB), Kanamycin (KAN), para-amino salicylic acid (PAS), levofloxacin (LFX), ethionamide (ETO), gatifloxacin (GAT), moxifloxacin (MOX), linezolid (LND), clarithromycin (CLA), azithromycin (AZM) and amikacin (AMK). Lineage data were available for 54/150 (36%) of the isolates (Supplementary Table 1). There were statistically significant differences (*p* = 0.00006) in the proportions of lineage 2 (East-Asian) isolates represented among groups; pan-S (3/7 isolates, 42.85%), pre-XDR (27/31 isolates, 87.1%) and XDR-TB (16/16, 100%).

### Overall metabolic profiles

The metabolites (n = 4071) of *Mtb* isolates with different susceptibility profiles are shown in Supplementary Table 2. The raw mass spectral data were processed by peak labeling, baseline filtering, retention-time correction, normalization and other standard procedures. In the positive and negative spectrum modes, 2526 and 1545 characteristic ion peaks were detected, respectively. Metabolites identified in both positive and negative modes were used for the downstream analysis. The standard quality-control (QC) strategy was applied, and their coefficient of variation (CV) is shown in Supplementary Table 3. The mean %CV across the 17 QC repeats showing the variation at 33.12%.

### Metabolomic comparisons among pan-S, pre-XDR and XDR-TB isolates

**Figure 1:**
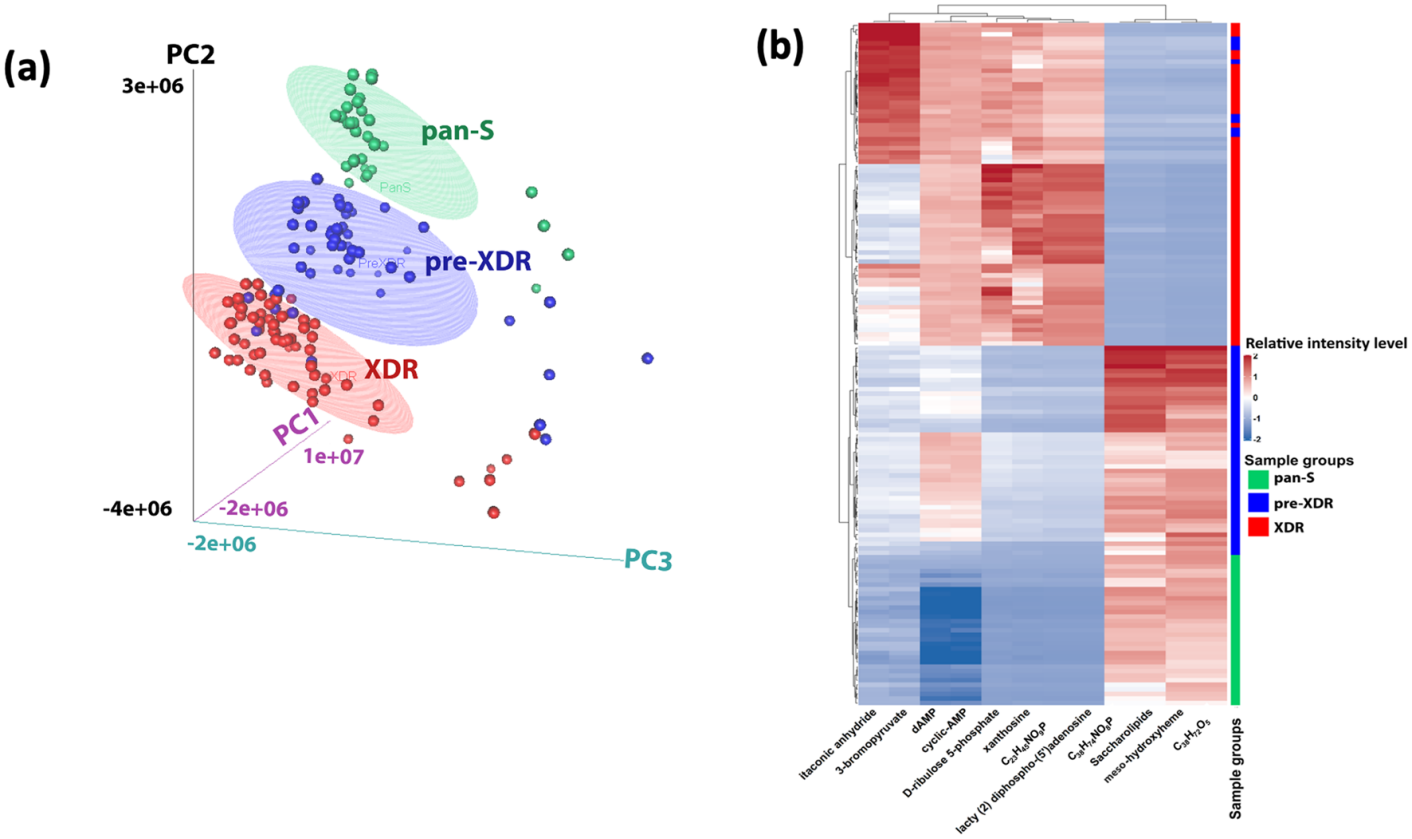
3D-PCA of pan-S, pre-XDR and XDR *Mtb* isolates (**a**). 3D-PCA was conducted to determine whether the groups could be distinguished based on metabolomics. There is a distinct separation between pan-S (green) and drug-resistant groups (blue and red). The pan-S *Mtb* isolates in this study can be separated from drug-resistant groups using metabolomic data. The heat map (**b**) shows the relative expression levels of the twelve metabolites with the greatest differences in levels among pan-S, pre-XDR and XDR-TB groups (n = 150 samples).

**Figure 2:**
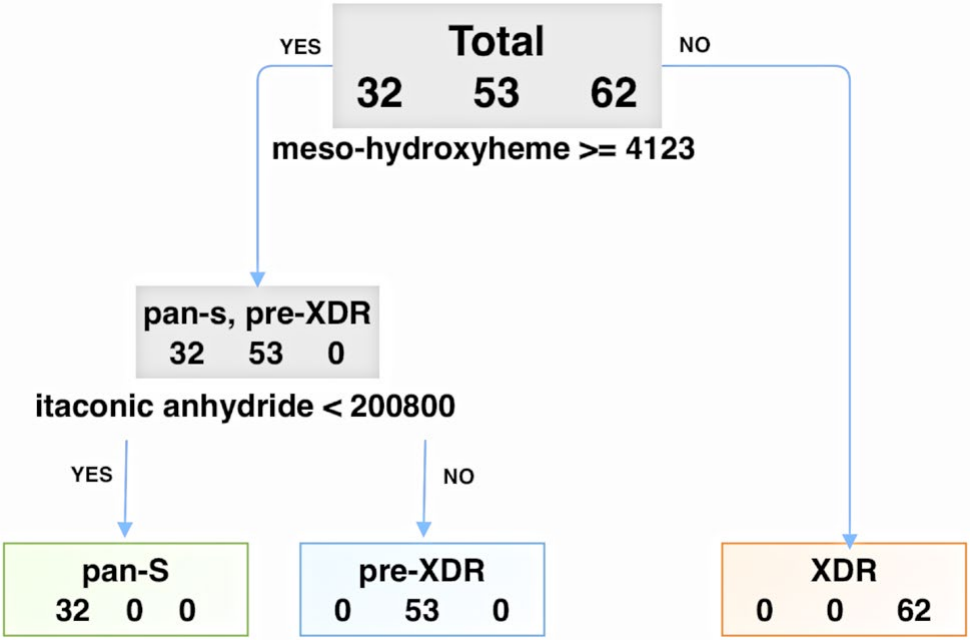
Decision tree for classification of drug-resistance status based on levels of two metabolomic markers (meso-hydroxyheme and itaconic anhydride). Sensitivity and specificity were both 100% for assignment of any sample to the correct group.

The patterns of metabolomes among pan-S, pre-XDR and XDR-TB isolates were analyzed using 3D-PCA. There was a distinct separation between pan-S (green) and drug-resistant isolates (blue and red) based on 3D-PCA (Fig. 1a). The top twelve metabolic markers for differentiation among pan-S, pre-XDR and XDR-TB groups are shown (Table 1). We then further analyzed the metabolomic pattern with heat-map analysis (Fig. 1b) using the twelve metabolites with the greatest variation between groups. The dendrogram shows the correlation between the relative intensities of metabolites in each sample. The decision tree for classification among pan-S, pre-XDR and XDR-TB groups is shown in Fig. 2. The probability of assigning each sample to the correct group was 100% (Table 2) and only two metabolites (meso-hydroxyheme and itaconic anhydride) were required. These two metabolites were not found in the human metabolite database (HMDB).

**Table 1:** The top twelve metabolic markers for differention among pan-S, pre-XDR and XDR-TB groups.

ID	Metabolites	Mean of intensity (arbitrary unit) of metabolites in each group			Between-group comparisons					
		pan-S	pre-XDR	XDR	pre-XDR vs pan-S		XDR vs pan-S		pre-XDR vs XDR	
					Fold change	p-values	Fold change	*p*-values	Fold change	*p*-values
1	itaconic anhydride	190,974.18	246,525.56	285,871.52	1.291	4.93E-12	1.16	3.73E-18	1.497	1.49E-04
2	3-bromopyruvate	221,172.55	286,524.57	330,349.71	1.295	1.38E-12	1.153	1.50E-18	1.494	1.80E-04
3	D-ribulose 5-phosphate	2,473.86	7,890.71	17,502.15	3.19	1.57E-13	2.218	4.45E-39	7.075	1.52E-23
4	xanthosine	2,917.40	9,131.20	21,779.42	3.13	2.42E-14	2.385	1.88E-46	7.465	1.97E-30
5	cyclic-AMP	247,799.09	822,971.75	977,998.24	3.321	2.43E-32	1.188	5.00E-28	3.947	6.78E-10
6	dAMP	47,267.80	171,137.06	203,507.92	3.621	3.06E-33	1.189	3.63E-29	4.305	1.79E-09
7	meso-hydroxyheme	21,779.08	23,788.05	1,710.21	1.092	1.46E-01	0.072	6.40E-30	0.079	8.24E-24
8	lacty (2) diphospho-(5') adenosine ^a^	143,104.96	594,299.48	1,691,809.62	4.153	1.94E-15	2.847	5.57E-57	11.822	1.45E-39
9	C_23_H_45_O_9_P^a^	143,104.96	594,299.48	1,691,809.62	4.153	1.94E-15	2.847	5.57E-57	11.822	1.45E-39
10	C_38_H_72_O_5_	21,779.08	23,788.05	1,710.21	1.092	1.46E-01	0.072	6.40E-30	0.079	8.24E-24
11	C_38_H_74_NO_8_P ^a^	19,035.57	20,742.42	1,434.37	1.09	2.36E-01	0.069	1.90E-24	0.075	6.67E-21
12	Saccharolipids ^a^	19,035.57	20,742.42	1,434.37	1.09	2.36E-01	0.069	1.90E-24	0.075	6.67E-21

pan-S, pan-susceptible; pre-XDR, pre-extensively drug-resistant tuberculosis; XDR-TB, extensively drug-resistant tuberculosis. ^**a**^Metabolites that have the same molecular weight and were differentially detected from the metabolomic analysis.

**Table 2.** The probability of predicting group membership for each isolate using a decision tree.

Reference isolates	Probability (%)			Predicted
	pan-S	pre-XDR	XDR-TB	
pan-S (n=33)	100	0	0	pan-S
pre-XDR (n=54)	0	100	0	pre-XDR
XDR (n=63)	0	0	100	XDR-TB

pan-S, pan-susceptible; pre-XDR, pre-extensively drug-resistant tuberculosis; XDR-TB, extensively drug-resistant tuberculosis

### O-PLS-DA among pan-S, pre-XDR and XDR-TB isolates

**Figure 3:**
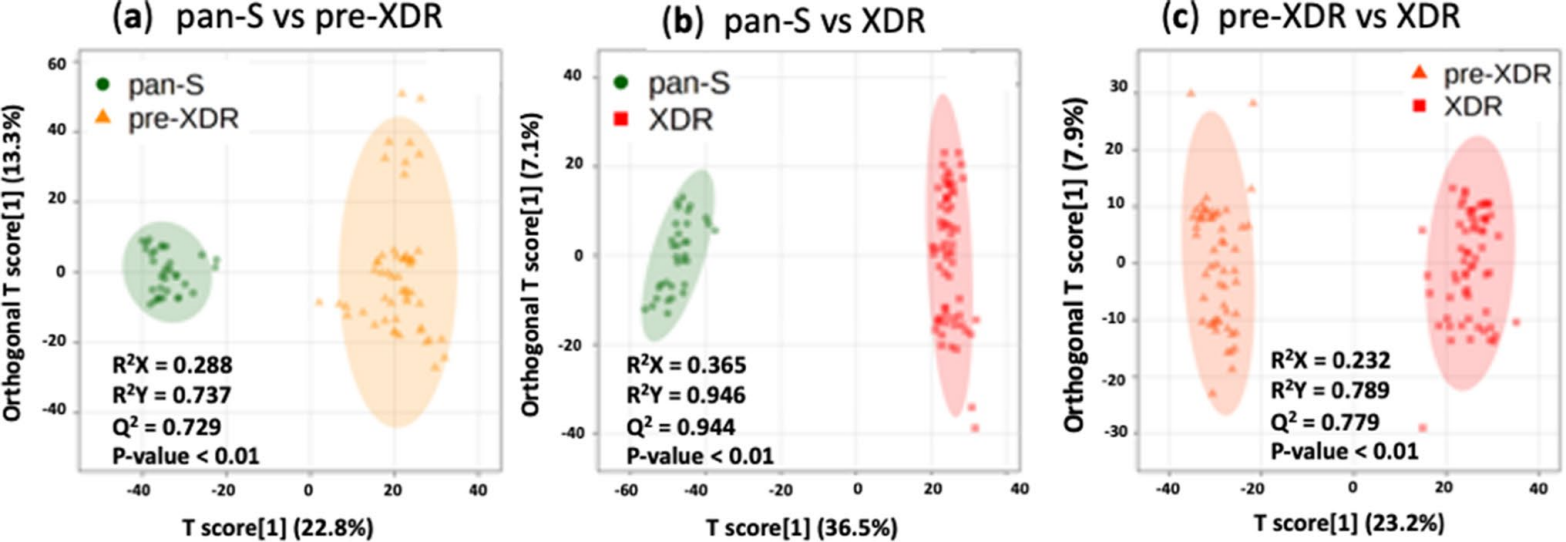
Orthogonal partial least-squares discriminant analysis (O-PLS-DA) cross-validated score plots showing significant separation between all pairs of groups; (**a**) pan-S vs pre-XDR, (**b**) pan-S vs XDR-TB and (**c**) pre-XDR vs XDR-TB. Green circles depict pan-S isolates, orange triangles depict pre-XDR isolates and red squares depict XDR isolates. All three comparisons show *p*-value < 0.01 and predictive abilities of (**a**) are (R^2^X = 0.288, R^2^Y = 0.737 and Q^2^ = 0.729) (**b**) are (R^2^X = 0.365, R^2^Y = 0.946 and Q^2^ = 0.944) and (**c**) are (R^2^X = 0.232, R^2^Y = 0.789 and Q^2^ = 0.779).

**Figure 4:**
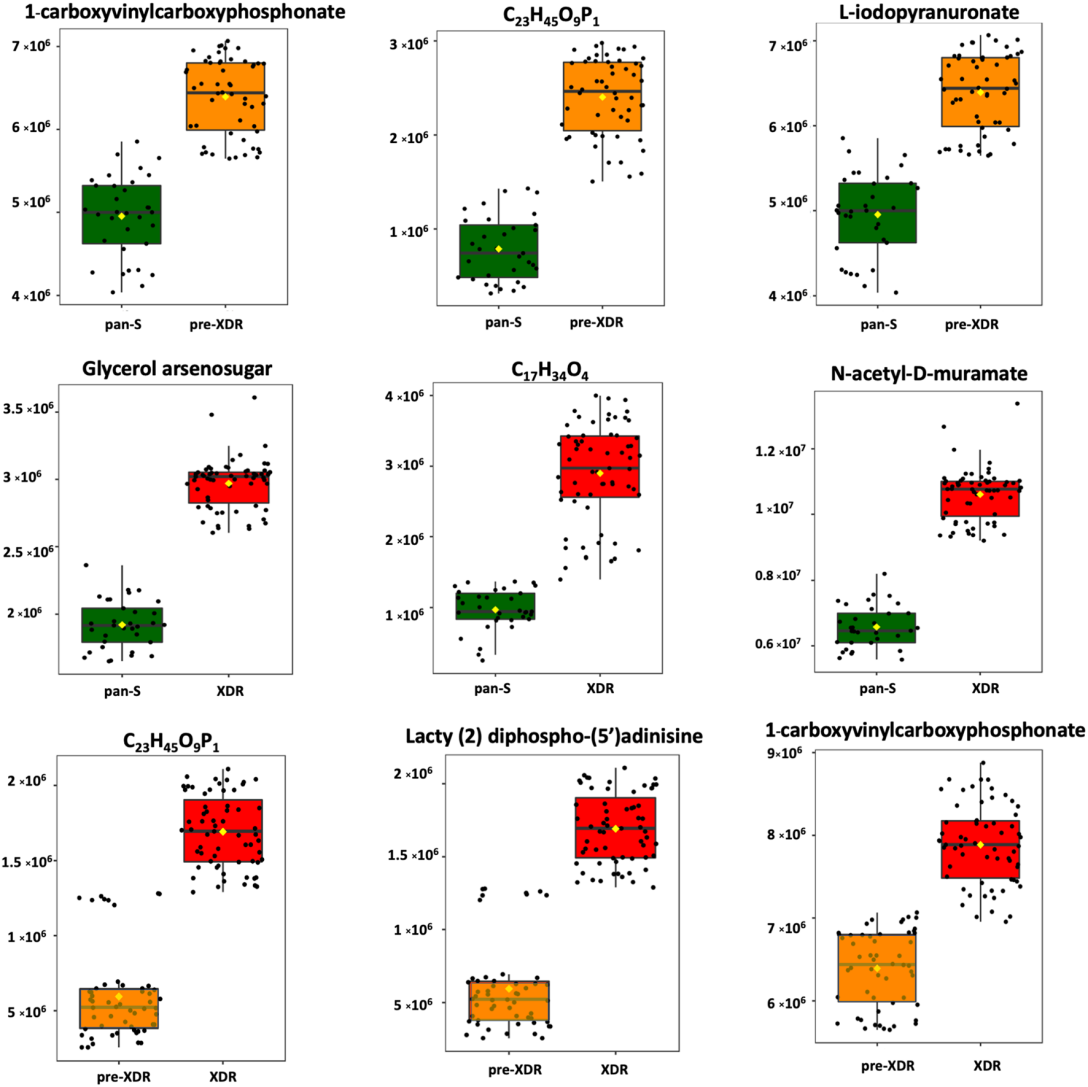
Metabolites differing significantly among pan-S, pre-XDR and XDR-TB groups based on O-PLS-DA. The green color shows the pan-S group, the orange color shows the pre-XDR group, the red color shows the XDR-TB group. Each black dot represents a single M. tuberculosis isolate. Only the top three markers in each pairwise comparison are shown.

Besides 3D-PCA, supervised O-PLS-DA was used for pairwise comparisons (pan-S vs. pre-XDR, pan-S vs. XDR-TB and pre-XDR vs. XDR-TB). The O-PLS-DA score plot showed a clear separation among the three *Mtb* groups (Fig. 3). When compared with pan-S, 1-carboxyvinylcarboxyphosphonate, C_23_H_45_O_9_P and L-iodopyranuronate are the most significantly increased metabolites in the pre-XDR group (Fig. 4, (upper row)). Glycerol arsenosugar, C_17_H_34_O_4_ and N-acetyl-D-muramate are the most significantly increased metabolites in the XDR-TB compared with the pan-S group (Fig. 4, (middle row)). Lacty (2) diphospho-(5′) adenosine, 1-carboxyvinylcarboxyphosphonate and C_23_H_45_O_9_P are the most significantly increased metabolites in XDR-TB compared with pre-XDR (Fig. 4, (lower row)).

### Metabolomic analysis of ethionamide- and ethambutol-resistant isolates

**Figure 5:**
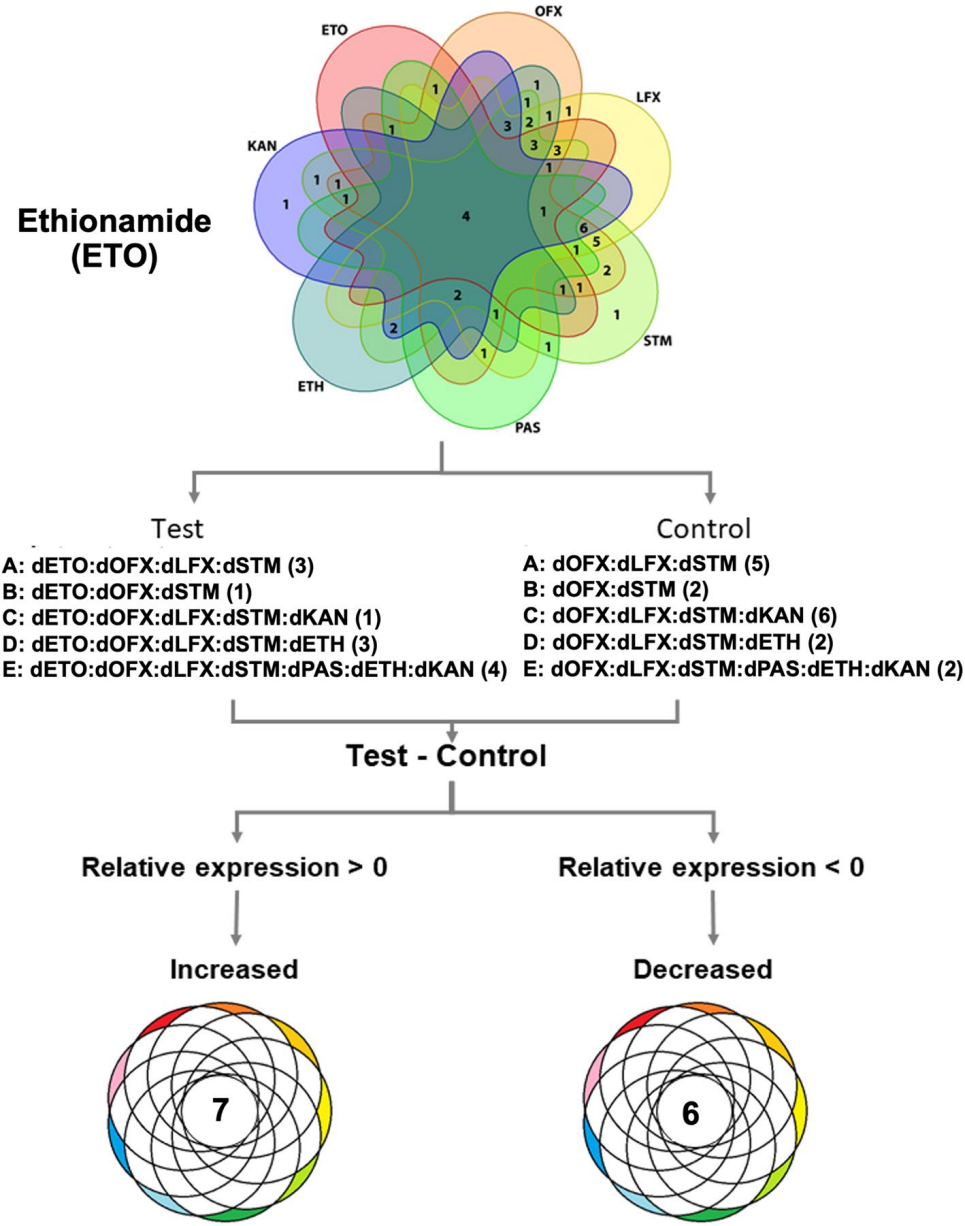
Metabolite markers to identify ethionamide resistance. The comparison is based on the five subsets of matched test and control subgroups (**A** to **E**) that contain various drug-resistance patterns. Only matched test strains (n = 12 from among ETO-resistant strains) and control strains (n = 17) were used. Then, a Venn diagram was created using the Venn function in R-programming. The specific controls (any isolates without ETO resistance) were used to create a new comparison. Metabolites present at higher or lower levels were analyzed after a comparison of metabolite expression levels between test and control. Only intersecting results found in all five comparisons were filtered. The numbers in brackets of each group refer to the number of isolates included. For ETO, seven increased and six decreased metabolites were identified.

To ensure that identification of the metabolic markers found in ETO- and ETH-resistant isolates were not confounded by lineage-specific factors, the proportion of each lineage was compared between the resistant and susceptible groups. There was no significant difference (*p* = 0.875) in the proportion of lineage 2 isolates that were ETO resistant (12/12 isolates with available lineage data) versus those that were ETO susceptible (15/17 isolates with available lineage data) (Supplementary Table 4). Seven metabolites had increased levels (N-acetyl-D-muramate, 2,4,6-trinitrobenzene sulfonate, C_24_H_50_N_1_O_7_P, glycerophospholipids, C_33_H_61_O_17_P, C_54_H_102_O_13_ and C_61_H_115_O_19_P) and six had decreased levels (L-histidinol phosphate, cyclic-AMP, 2-iodophenol, 6-deoxy-5-ketofructose-1-phosphate, glycerol arsenosugar and fatty acyls) relative to ETO-susceptible isolates (Fig. 5 and Supplementary Table 5).

**Figure 6:**
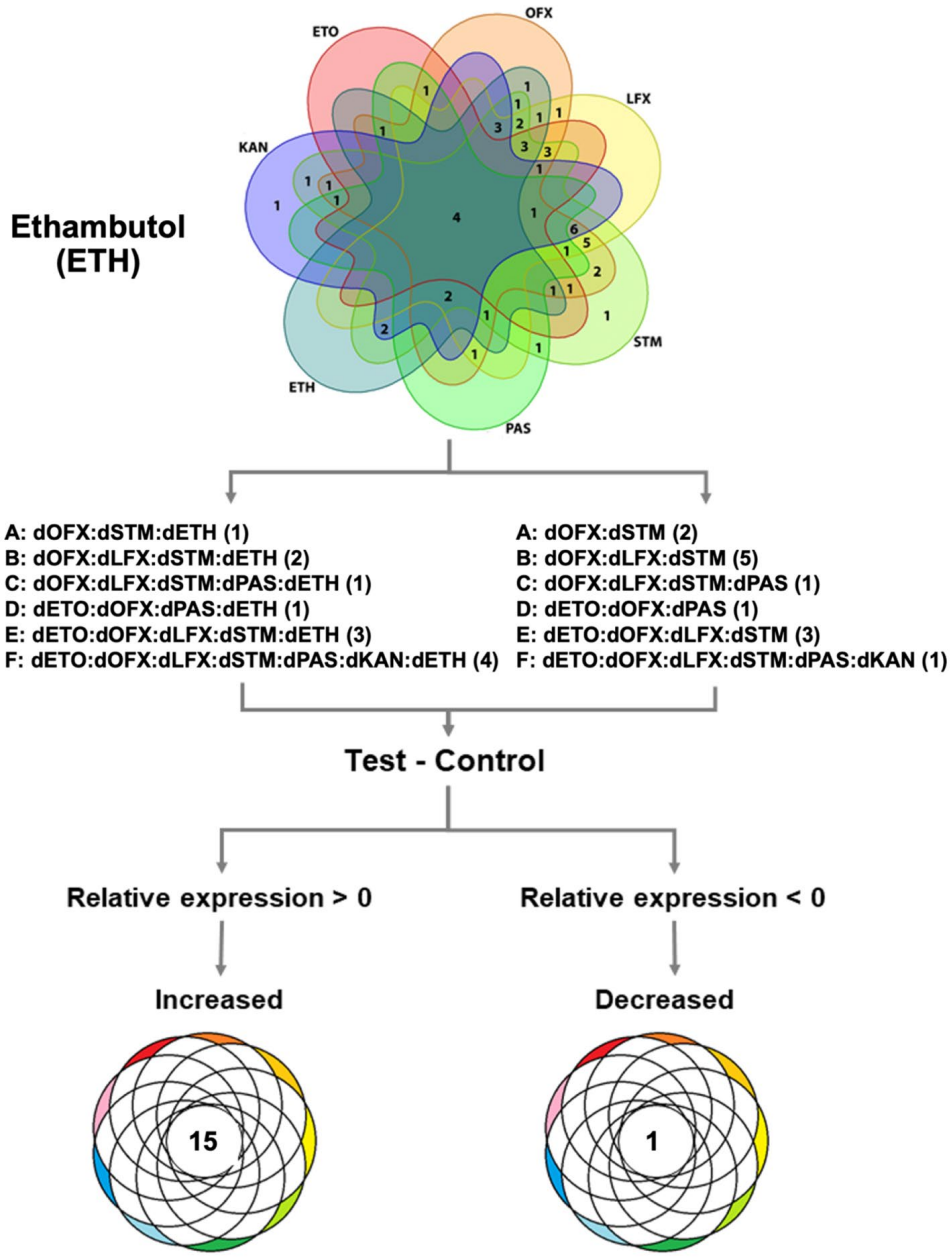
Metabolite marker to identify ethambutol (ETH) resistance. The comparison is based on each of the six subsets of the matched test and control (**A** to **F**) that contain various drug resistance patterns. Only the matched Test and Control; Test (n = 12 from 17 ETH resistant strains) and Control (n = 13) were used. The subset of isolates with (test) and without (control) ETH phenotypic resistance was filtered. Then, a Venn diagram was created using the Venn function in R-programming. The specific controls (any isolate without ETH resistance) were used to create a new comparison. Metabolites present at higher or lower levels were analyzed after a comparison of metabolite expression levels between test and control. Only intersect results found in all six comparisons were filtered. The numbers in the brackets of each group confer to the number of isolates. For ETH, fifteen increased and one decreased metabolite were found.

Similarly, there was no significant difference (*p* = 0.905) in the proportion of lineage 2 isolates that were ETH resistant (11/12 isolates with available lineage data) versus those that were ETH susceptible (13/13 isolates with available lineage data) (Supplementary Table 4). Metabolomic analysis of ETH-resistant isolates revealed 15 metabolites with increased levels; O-acetyl-L-homoserine, (indol-3-yl)pyruvate, (7,8-dihydropterin-6-yl) methyl diphosphate, (D-alanyl)adenylate, S-(hydroxymethyl) bacillithiol, 2,4-dichlorotoluene, 3-bromopropanesulfonate, metosulam, C_19_H_38_O_4_, C_21_H_42_O_4_, C_27_H_54_O_2_, gycerophospholipids, C_21_H_44_N_1_O_7_P, C_76_H_146_O_6_ and glycerolipids (Fig. 6 and Supplementary Table 6). The level of one metabolite (bromoacetate) was lower in ETH-resistant isolates.

## Discussion

Only one previous study has investigated metabolic markers of *Mtb* that might distinguish pan-S (n = 18), MDR (n = 17) and XDR (n = 18) isolates^16^. That study used a relatively small sample size and did not identify any significant metabolites that could differentiate between their MDR and XDR isolates. Another study investigated TB-patient serum metabolomics and discovered four potential biomarkers: N1-methyl-2-pyridone-5-carboxamide (N1M2P5C), 1-myristoyl-sn-glycerol-3-phosphocholine (MG3P), caprylic acid (CA), and D-xylulose (DX) that, in combination, could discriminate between MDR-TB (n = 30) and pan-S (n = 30) with both sensitivity and specificity of 86.7%^8^. Here, we analyzed metabolomic profiles of *Mtb* isolates of known drug-resistance status to differentiate between pre-XDR and XDR-TB. We also specifically analyzed the metabolic pattern of isolates resistant or sensitive to ETO and ETH, two drugs for which there is frequently a discrepancy between genotypic and phenotypic DST results^5,17,18^.

We expected that *Mtb* isolates with different drug-susceptibility profiles would be metabolically diverse, and that untargeted metabolomics should show metabolic patterns correlated with drug susceptibility or resistance. Based on 3D-PCA, clear differentiation between the pan-S and drug-resistant isolates (pre-XDR and XDR-TB) was found. However, the pre-XDR and XDR-TB isolates were not totally separated. Then, we used a decision tree as an approach to differentiate among the three TB groups. Interestingly, only two metabolites (meso-hydroxyheme and itaconic anhydride) were required to provide 100% sensitivity and 100% specificity for distinguishing among the three groups. Meso-hydroxyheme is a key intermediate of the *Mycobacterium* heme utilization degrader (MhuD) reaction^19^. MhuD converts host-derived heme into iron by degrading it^19–21^. Iron is required for numerous essential biological processes and is associated with the aminoglycoside-resistance mechanism of *Mtb*^22^. Iron and heme are thought to be potential targets for future drug development due to their uptake into pathogens^23^. The higher level of meso-hydroxyheme in resistant isolates could explain the difference among drug-resistance types. However, our analysis revealed that XDR-TB isolates had lower meso-hydroxyheme compared to pan-S and pre-XDR. Possibly, XDR-TB isolates require less iron for survival than pan-S and pre-XDR isolates. The limiting of available iron in the human host, which is sequestered in high-affinity binding proteins such as heme, is an important part of the innate immune response to bacterial infections^24^. Lowering the amount of iron required for survival in the host could be one of the adaptations of XDR-TB isolates. Another metabolite that was differentially expressed among DR-TB types is itaconic anhydride. This metabolite is an inhibitor of isocitrate lyase, a key enzyme that enables the bacilli to persist under oxidative-stress conditions^25^. Possibly, a drug-resistant isolate is more fit to survive in hostile environments^26^. Neither of these two metabolites has any matches in the human metabolic database. Therefore, besides being diagnostic markers for DST, these metabolites might potentially find a use in treatment monitoring to detect any change of drug susceptibility of the pathogen in patients. To confirm the clinical application of these markers, further study investigating these metabolites in patient serum during the course of treatment is needed.

We also used O-PLS-DA for pairwise analyses among the three groups (pan-S, pre-XDR and XDR-TB) to identify metabolic markers. All pairwise comparisons yielded clear distinctions, so we focused on the three most-significantly increased metabolites in each case. Interestingly, levels of C_23_H_45_O_9_P differentiated between pre-XDR and pan-S and also between pre-XDR and XDR-TB isolates. This metabolite therefore shows great potential for discrimination among drug-resistance types. The metabolites identified as important using O-PLS-DA differed from those identified using the decision tree because the two methods use different algorithms: one identifies markers that can be used to differentiate among three groups whereas the other makes only pairwise comparisons. Nonetheless, based on the metabolic patterns that are clearly different between groups, the potential applicability remains for the metabolome to distinguish isolates with different drug-resistance properties.

Phenotypic DST of *Mtb* for certain drugs can be problematic due to the uncertain MIC cut-off values for some drugs and the extent of reproducibility of such tests^27^. Previously, our research group reported that 65% of isolates that were phenotypically ETH resistant and 62% that were phenotypically ETO resistant did not yield the same results according to genotypic resistance analysis^5^. Phenotypic drug-resistance profiles are not always associated with distinctive metabolic fingerprints^16^. Rego et al. (2021) compared a relatively low number of *Mtb* isolates (n = 53) and did not investigate specific drugs, especially ETH and ETO. In our study, we attempted to identify the metabolomic patterns associated with phenotypic resistance against ETH and ETO using subsets of DR *Mtb* isolates. Due to the high level of genotypic /phenotypic discrepancy for ETO and ETH (Supplementary Table 4), we relied on the gold standard of drug-susceptibility testing using the agar proportion method^28^.

We analyzed the relative amounts of metabolites in ETO-resistant isolates (that were also resistant to other drugs) and subtracted from these the expression levels in isolates that were not resistant to ETO (but some may have been resistant to other drugs). Only the common metabolites found in ETO-resistant isolates and not in any ETO-susceptible isolates were counted as ETO resistance-specific metabolites. We found seven increased and six decreased metabolites specific to ETO-resistant isolates. Changes in these metabolites might be associated with the ETO-resistance mechanism*.* The most increased metabolites included glycerophospholipids (GPL) and C_61_H_115_O_19_P. The literature suggests that the bacterial stress sensor may respond directly to GPL concentration^29^. Previously, comparative lipidomic analysis revealed an increased amount of fatty acyls and GPL in DR-TB: both metabolites are important for *Mtb* virulence and pathogenicity^30^. The most important metabolite showing decreased levels in ETO-resistant isolates was cyclic-AMP (cAMP). A variety of cyclic nucleotides are utilized by *Mtb*, including cAMP, cyclic-di-AMP (c-di-AMP) and cyclic-di-GMP (c-di-GMP) that regulate bacterial cell physiology and disrupt signaling in host cells^31^. The cAMP is important for gene regulation in mycobacteria, and the ability to secrete cAMP into host macrophages during infection contributes to *Mtb* pathogenesis^32^. C-di-GMP has been reported to promote the resistance of *Mtb* to ETH, possibly because increased Ethr activity suppresses *ethA* expression, lowering the amount of active ETH in the bacterial cytoplasm^33^. However, the role of cAMP in the drug-resistance mechanism in *Mtb* is unknown and needs to be further investigated*.* Most of the ETH- and ETO-resistant isolates used in our study belonged to lineage 2. This lineage is strongly associated with MDR-TB phenotypes^34,35^ and acquisition of resistance^34^. Due to the lineage 2 proportion between ETH/ETO resistant and susceptible isolates are relatively comparable, the metabolomic markers we found is therefore not confounded by the lineages of *Mtb*.

We used a similar approach to identify the metabolites associated with ETH resistance. In ETH-resistant isolates, fifteen metabolites exhibited increased expression levels and only one had decreased levels. The most increased metabolites included (indol-3-yl)pyruvate and 3-bromopropanesulfonate. The latter is a specific inhibitor of methyl-CoM reductase and completely inhibits dechlorination of 1,2-DCA but has not previously been considered in the context of drug resistance^36^. The most decreased metabolite was bromoacetate, used by researchers as a toxic small molecule to model the selective pressures imposed by antibiotics and anthropogenic toxins in *Escherichia coli.* Further study of these changed metabolites is needed to explain the ETH-resistance mechanism of *Mtb.* A previous study used untargeted urine metabolomics with gas chromatography-time of flight mass spectrometry (GC-TOF–MS) to investigate the drug metabolism of a TB patient cohort (n = 20)^37^. They identified 2-aminobutyric acid (AABA) as the specific metabolite associated with ethambutol resistance. However, AABA was not on our list of ETH-resistance metabolites. This might be due to differences in study design, especially given that our model investigated pathogen metabolites compared to human metabolites.

Like ETO resistance-associated metabolites, no information is available on whether the metabolites specifically found in ETH-resistant isolates are actually associated with mechanisms of resistance. Although statistically significant, changes in metabolite levels in each group were usually less than two-fold, which might or might not be biologically meaningful and/or reproducible. Additional studies are needed to fill this knowledge gap.

In laboratory diagnosis, pathogen detection using microscopic and/or molecular techniques including drug-susceptibility tests are used to identify DR-TB^38^. However, these techniques are laborious, and the DST results are sometimes discordant between methods. In the advanced “omics” era, our findings might support the future development of metabolomics-based TB diagnosis. In a clinical setting, the metabolite patterns of *Mtb* could also be useful. Acquired resistance might occur, defined as resistance to one or more anti-TB drugs in isolates recovered from patients who had received previous anti-TB treatment^39^. In acquired resistance, metabolomics of *Mtb* can change according to the resistance phenotype^40^. Here, we focused on untargeted LC–MS-based metabolomics. The major advantage of this approach is the discovery of novel metabolites in relation to the study context. We showed that metabolomics of *Mtb* could be used to distinguish between various DR-TB strains as well as between isolates that were phenotypically resistant to ETO and ETH. However, the reproducibility of the metabolomic analysis from the machine is still one of the concerns as reflected with %CV over 30% in the QC sample set. This technical limitation could be managed by repeated sampling, an approach which would increase the cost per test for an already expensive technique. Therefore, the application of metabolomics as a diagnostic aid for personalized treatment and monitoring of TB patients is still limited due to the high cost, low reproducibility, and requirement for sophisticated equipment. As with other advanced methods, such as high-throughput DNA sequencing, we can expect the cost of mass spectrometry to decrease in the future, making it suitable for affordable and routine use. Much more research and development are needed to make this technology cost effective, easy to use and practical in real-world settings. The work reported here has laid the foundation for further study and validation.

Limitations of our study should be discussed. We analyzed only pre-XDR and XDR-TB strains compared to pan-S. As MDR-TB *Mtb* isolates are resistant to INH and RIF, a state that is shared by pre-XDR and XDR-TB, we included only highly resistant isolates in the study. We assumed that metabolomics changed more as resistance increased. No clinical data such as the treatment regimens of TB patient were available. The proportions of isolates of different lineages among groups were significantly different. Therefore, the unequal representation of lineages might have influenced the metabolite markers found. Additional study controlling for lineage before testing drug-resistance pattern should be done. For some *Mtb* isolates, there were no available phenotypic DST data. Therefore, these isolates could not be included for metabolomic analysis relating to ETO and ETH resistance. Although the sample size for ETO and ETH metabolomic analysis was limited, lineage representation did not significantly differ among the datasets used and the analysis approach was very stringent, giving us confidence in the findings. Because of the limited number of samples available, a testing/validation set analysis approach could not be used. Therefore, the random sampling approach indicating 100% accuracy might not truly reflect the actual performance of metabolomic analysis for DR-TB identification. The metabolomic data used in this study was from normal, cultured cells that were not subject to stresses such as presence of drugs. To increase the safety of the analysis protocol, we used only dead bacterial cells for sample preparation. We avoided experiments that included activation of the bacteria with anti-TB drugs to stimulate the expression of bacterial metabolites.

The WHO Laboratory Biosafety Manual, has categorized drug-susceptible, drug-resistant, and multidrug-resistant *Mtb* strains into biohazard risk category 3 whereas XDR *Mtb* strains were assigned to risk group 4, the highest risk category for human and community health^41^. We used heat inactivation of the bacteria to ensure biosafety while working with XDR *Mtb* strains and applied the same method to all other strains for consistency. However, this approach could potentially alter the metabolome of bacteria subjected to heat and also cause the degradation of some metabolites. Therefore, the metabolomic analysis of heat-inactivated *Mtb* might not accurately reflect the normal metabolism and might give misleading information on the biology of *Mtb*. This is the key limitation of our study that should be considered when interpreting the results. Metabolomic profiles obtained following extraction methods that do not use heat inactivation^42^ should be investigated in the future.

The high number of bacterial cells obtained in culture yields a high concentration of metabolites, thus increasing the sensitivity for UHPLC-ESI-QTOF-MS/MS detection and analysis. However, it is uncertain that large amounts of these same metabolites would be released in clinical samples. The significant metabolomic markers we found were less than two-fold different between test and control groups. It is unclear whether this difference between the *Mtb* groups is biological meaningful and/or clinically significant. This needs to be further investigated. We attempted to analyze the metabolites associated with para-aminosalicylic acid resistance, but too few resistant and control isolates were available to reveal any significant metabolite marker.

In conclusion, we reported that *Mtb* metabolomics could distinguish among pan-S, pre-XDR and XDR according to levels of two metabolites (meso-hydroxyheme and itaconic anhydride). We also demonstrated the potential for metabolomics of *Mtb* to differentiate between isolates that were phenotypically resistant to ETO and ETH.

## Methods

### *Mtb* isolates and setting

*Mtb* isolates from our previous project^43^, for which phenotypic/genotypic drug-susceptibility results were available (total n = 150; 33 pan-S, 54 pre-XDR and 63 XDR-TB), were used in this study. The previous definition of XDR-TB, (multidrug resistant-TB (MDR-TB)) was that such isolates were resistant to any fluoroquinolone and at least one of three second-line injectable drugs (capreomycin, kanamycin and amikacin)^44^. We used this definition to classify TB groups in our previous drug-susceptibility test (DST) system. These isolates were collected from 1998 to 2013 from various provinces in Thailand. From 150 isolates, whole-genome sequence datasets were available for 54 isolates^43,45^ and lineage classification of these was done using RD-Analyzer^46^. Notably, some isolates exhibit resistance to more than one drug. This study used bacteria cultured from stored stock. Each *Mtb* isolate was cultured on Lowenstein-Jensen medium at 37 °C for 4 weeks, multiple colonies were scraped from the tubes, resuspended in sterile distilled water, stored at − 70 °C and material for the metabolomic analysis was extracted on the next day. Each sample was associated with information including the UHPLC-MS/MS-based metabolomics data (.d files) but without information that could lead to identification of any participant: no informed consent was required.

### Drug-susceptibility testing

Phenotypic DSTs for anti-TB drugs were performed using the standard agar proportional method^47^. Drug critical concentrations (CCs) used were 0.2 mg/L for isoniazid, 1.0 mg/L for rifampicin, 5.0 mg/L for ethambutol and ethionamide, 6.0 mg/L for amikacin and kanamycin, and 2.0 mg/L for streptomycin, *p*-amino salicylic acid, ofloxacin, levofloxacin, moxifloxacin and gatifloxacin. A critical proportion value of 1% was used. A culture that had **1%** or more growth on the medium containing the critical concentration of the anti-TB drug is considered as resistant**.** Genotypic drug susceptibility test results of ETO and ETH was based on our previous study^5^ that analyzed using TB-profiler^48^.

### Sample preparation

Stock cultures of *Mtb* inactivated by heat at 95 °C for 30 min were used. Colonies were suspended in HPLC grade water, and optical density was adjusted at 600 nm to ODs equal to 5, and 1500 μL of the resulting bacterial suspension were used. The metabolomics extraction was performed following the protocol of P A. Vorkas *et al.*^49^. Ten microliters aliquots from each sample were pooled and mixed in a 1.5 ml tube to make the quality-control (QC) sample and 120 µl of this was transferred to an HPLC glass insert.

### UHPLC-ESI-QTOF-MS/MS analysis

The aqueous-phase extracts of each sample were analyzed on a reverse-phase platform. The separation part was performed using the UHPLC system (Bruker, Germany) Bruker intensity solo HPLC C18 2.1 × 100 mm, 2 μm column (Bruker, Germany). The column temperature was set at 40 °C and the autosampler temperature was set at 4 °C. Mobile phase A was water 100% with 0.1% formic acid (FA) and mobile phase B was acetonitrile 100% with 0.1% FA. The flow rate was set at 0.35 ml/min and the elution gradient was set as follows: 99% A (0.0–2.0 min, 0.25 ml/min), 99–1% A (2.0–17.0 min, 0.25 ml/min), 1% A (17.0–20.0 min, 0.25 ml/min), 1–99% A (20.0–20.1 min, 0.25–0.35 ml/min), 99% A (20.1–28.3 min, 0.35 ml/min), 99% A (28.3–28.5 min, 0.35–0.25 ml/min), 99% A (28.5–30.0 min, 0.25 ml/min). Injection volume of sample (7 μl) was applied for both positive and negative ionization polarity modes. The mass spectroscopy part was performed using the compact ESI-Q-TOF system (Bruker, Germany). Sodium formate (2 mM sodium hydroxide, 0.1% FA, 50% isopropyl alcohol) was directly injected as an external calibrant with flow rate 0.5 μl/min. The condition in positive ionization polarity mode: mass range 50–1300 m/z, cone voltage 35 V, capillary voltage 4500 V, source temperature 220 °C, desolvation temperature 220 °C, desolvation gas flow 8 L/min. The conditions in negative ionization polarity mode: m/z range: 50–1300 m/z, cone voltage 31 V, capillary voltage 4500 V, source temperature 220 °C, desolvation temperature 220 °C, desolvation gas 8 L/min. The standard QC strategy was applied for the UHPLC-MS analysis. A pool of all samples was prepared as for QC. This QC sample was injected at the beginning, following every 10 sample injections, and at the end to estimate the instrument stability and determine reproducibility. Following sample analysis, QC sample dilutions, 1:2, 1:4, 1:8 and 1:16 in the reconstitution buffer, were run in the MS/MS mode, followed by extraction of a blank sample and reconstitution blank in MS mode to estimate the complements and impurity of the extraction and reconstitution solvent. The UHPLC-MS/MS-based metabolomics dataset is shown in Supplementary Table 2.

### Data analysis

**Figure 7:**
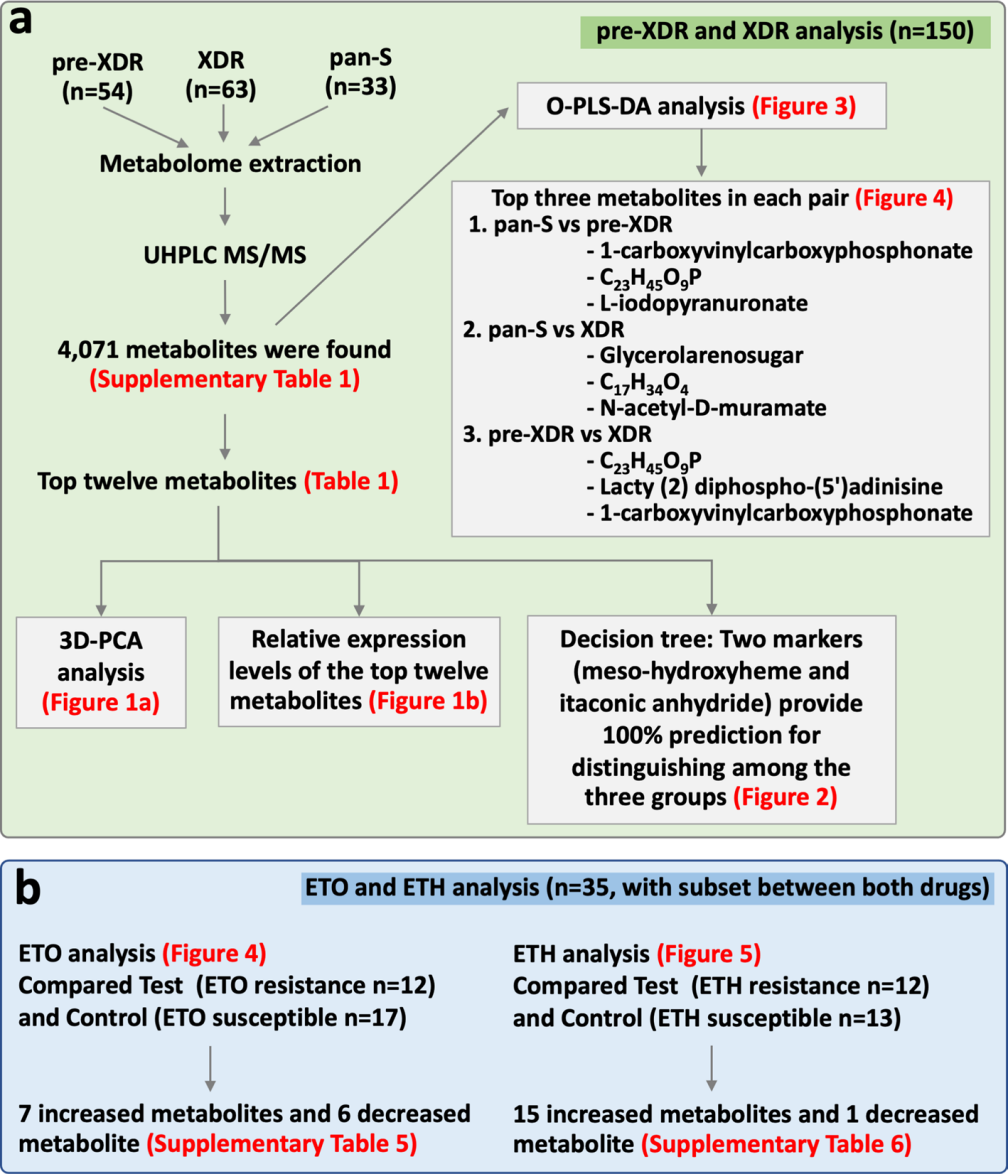
Study design and flow of metabolomic analysis. The study included two parts; analysis among pan-S, pre-XDR and XDR-TB isolates (**a**) and analysis of metabolite markers for identifying ETO/ETH resistance (**b**). Red letters depict the relevant figure and table results obtained in each step. Pan-S; pan-susceptible, pre-extensively resistance; pre-XDR, extensively resistance; XDR, Ultra-high performance liquid chromatography tandem mass spectrometry; UHPLC MS/MS, Orthogonal partial least-squares discriminant analysis; O-PLS-DA.

The metabolomics data from 150 *Mtb* samples were analyzed. CompassXport.exe v3.0.9.2 was used to convert data to .mzXML format. R-program version 4.1.2 (https://cran.r-project.org) was used in this study. The R-program library “faahKO” was used to convert mzXML to Computable Document Format (CDF)^50^. The sample-processing function of the MAIT package was applied to take a set of files containing LC/MS sample data and perform peak detection, retention-time correction and peak grouping^51^. Following this, the peakAnnotation function was used as spectra constructor and peak annotator. Accurate m/z (< 5 ppm) measurements of detected chromatographic peaks were first matched to metabolites from online MS databases (*Mycobacterium*_lipid^3^, *Mycobacterium* metabolite_Mycomass database^3^, *Mtb* database^52^ and NTM metabolite_BIOCYC database^52^). Study design and flow of the metabolomic analysis is shown (Fig. 7).

To distinguish among pan-S, pre-XDR and XDR-TB isolates based on metabolomics, after cleaning the data and identification of the metabolites, all statistical analysis including multidimensional statistical analysis, (a) intensity heat map (b) 3D principal-component analysis (3D-PCA) (c) relative-intensity box plot (d) and (e) decision-tree analysis, were performed using R-program. Jackknife sampling technique was used for the performance analysis, we sampled one isolate from each group then trained the remaining samples, pan-S (n = 32), pre-XDR (n = 53) and XDR-TB (n = 62) groups, and repeated this step for all samples in each group. We then compared metabolic markers with the human metabolomics database (HMDB). The decision trees for classification among pan-S, pre-XDR and XDR-TB were generated using rpart (R-program)^53^.

Orthogonal partial least-squares discriminant analysis (O-PLS-DA) was also used for classification among groups. The dataset of samples arranged in columns (pan-S, pre-XDR and XDR-TB) and variables in rows (metabolite intensities) was prepared as a .CSV file and the O-PLS-DA score was calculated and visualized using Metaboanalyst 5.0 (http://www.metaboanalyst.ca/faces/home.xhtml)^54^. Chi-square or Fisher's exact test was used for comparisons of lineage proportions between groups.

For ETH (or ETO) metabolic markers, the subset of isolates with (test) and without (control) ETH (or ETO) phenotypic resistance was filtered. Then, a Venn diagram for subset analysis was created using the Venn function in R-programming. The specific controls (any isolates without ETH (or ETO) resistance) were used to create a new comparison. Metabolites present at higher or lower levels were analyzed after a comparison of metabolite expression levels between test and control.

### Ethical approval

The study protocol was approved by the Center for Ethics in Human Research, Khon Kaen University (HE601249).

## Supplementary Information

Supplementary Tables.

## Data Availability

The datasets generated and analyzed during the current study are available in the MassIVE repository with accession number MSV000091354, [https://massive.ucsd.edu/ProteoSAFe/dataset.jsp?accession=MSV000091354].
